# Conditions for distributed leadership practices among managers in elder- and disability care organizations: A structural equation modeling approach

**DOI:** 10.1016/j.ijnsa.2021.100049

**Published:** 2021-10-20

**Authors:** Caroline Hasselgren, Lotta Dellve, Gunnar Gillberg

**Affiliations:** a Department of Sociology and Work Science, University of Gothenburg, Sweden; b AGECAP – Centre for Ageing and Health, University of Gothenburg, Sweden

**Keywords:** Distributed leadership, Eldercare, Disability care, First line managers, Work team relations, Trusting collaborations, Structural equation modeling, Public sector, Sweden

## Abstract

**Background:**

Against the backdrop of worldwide increases in life expectancy, there is a growing concern about the future of health and social care services in many countries, including Sweden. This is not least due to expected cutbacks in tax revenues and increasing staff shortages in the welfare sector. Challenges such as these have spurred leadership reforms aimed at mobilizing employee engagement and promoting job attractiveness. For instance, distributed leadership, whereby leader responsibilities are distributed and shared among team members, has gained momentum in recent decades. Nevertheless, there is still limited knowledge as to whether and how organizational conditions impact first-line managers’ inclination to distribute influence and control.

**Objective:**

This study aims to examine the organizational precursors of two interrelated aspects of distributed leadership among first-line managers in municipal elder- and disability care: collaborative decision-making and the presence (or lack of) a participatory leadership approach.

**Methods:**

Utilizing survey data from managers working in the elder- and disability care sectors in the municipality of Gothenburg, Sweden (*N* = 250), associations between conditions and aspects of distributed leadership were analyzed by means of Confirmatory Factor Analysis (CFA) and Structural Equation Modeling (SEM).

**Results:**

Both aspects of distributed leadership were positively and significantly associated with managers’ perceptions of having well-functioning collaborations with their employees (*β* = 0.277 [0.122–0.432]; *β* = 0.492 [0.346–0.637]) as well as with the managers’ active participation in development work aimed at, e.g., promoting organizational trust (*β* = 0.242 [0.039–0.446]; *β* = 0.251 [0.103–0.398]). No significant associations between distributed leadership and support from senior management or positive collaborations with support functions were observed in the controlled analyses. However, managers’ perceptions of organizational governance deficits were shown to be significantly and positively associated with having a more participatory leadership approach (*β* = 0.261 [0.032–0.491]).

**Conclusions:**

In line with the notion of distributed leadership as a “collective activity”, which is realized in the interaction between managers and their employees, the findings demonstrate that trusting collaborations with responsible and knowledgeable employees play a key role in its practical implementation. Also, distributed leadership, and the work team relations through which it is enabled, are likely to mutually and positively reinforce each other. Additionally, the analyses revealed that managers’ experiences of poor organizational governance appear to promote certain distributed leadership practices. Potentially, this could be because such deficits encourage them to seek support and guidance from their employees, but more research exploring these mechanisms is needed.

## 1 Introduction

Against the backdrop of rapid increases in life expectancy and complex comorbidities in older populations, the pressure on healthcare systems is increasing worldwide and elder- and disability care services are expanding. In many countries, there is thus a growing concern about the future availability of financial resources and trained staff in these areas (see, e.g., Blank et al. 2017, Fujisawa and Colombo 2009, Gusmano and Okma 2018, OECD 2020, SALAR, 2018, Scales 2021). In Sweden, for instance, the welfare sector suffers from high levels of sick-leave and employee turnover, and staff shortages are expected to increase extensively in the coming years (SALAR, 2018, 2019). This implies that, among other things, organizational resource mobilization efforts must include measures to promote job attractiveness, engagement, and sustainable working conditions for trained staff. Many initiatives have so far been tried but few have managed to achieve any noticeable results in practice. Organizational gaps, managers’ work overload and poor functional support have been identified as important obstacles for making necessary improvements (Dellve and Wolmesjö, 2016; Gillberg and Dellve, 2019). Moreover, the implementation of overly controlling top-down initiatives has spurred decreased engagement, frustration, stress and health problems among both managers and employees (Dellve et al., 2018; Fallman et al., 2019).

Among other things, challenges such as these have increased scholars’ and practitioners’ interest in new forms of leadership. For instance, many have suggested that a shift away from more traditional views, in which the attributes of individual leaders are the main focus, is warranted. In line with this, successful leadership is now increasingly recognized as a group-level phenomenon (Nielsen and Daniels, 2012) and more “collectivistic” approaches, whereby leader responsibilities are distributed and shared among team members, have gained particular momentum in recent decades (see, e.g., Beirne 2017, Currie and Lockett 2011, De Brún et al. 2019). In fact, such approaches are assumed to be particularly “desirable in public services because [they are] inclusive and alig[n] with recent organizational restructuring towards the flatter organization” (Currie and Lockett, 2011). In health and social care contexts specifically, more collectivistic forms of leadership are expected to enhance “organizational resourcefulness” (Gronn, 2015) by, for instance, having a positive impact on the quality and safety of care (Boak et al., 2015; Greenfield et al., 2009; McKee et al., 2013) as well as by improving employee commitment, satisfaction and overall team performance (De Brún et al., 2019; Quek et al., 2021). Although Swedish organizations have a long tradition of safeguarding values such as consensus, employee influence, delegation and open dialogue between levels and parties (Sandberg and Movitz, 2013), contemporary policy changes aimed at implementing so-called trust-based governance in the public sector testifies to attempts of reforming leadership even further in accordance with more collectivistic ideals (Ministry of Employment, 2018).

This study seeks to contribute to the field by specifically focusing on distributed leadership (DL), which is one example of a more collectivistic approach to leadership. More specifically, it examines the enactment of DL, as well as its potential precursors, in municipal elder- and disability care organizations in Sweden. Thereby, it offers further insights as to whether and how DL is configured through an interaction between first-line managers (i.e., unit managers and group leaders), their employees and the organizations’ overall management and support resources.

### 1.1 Distributing leadership – a definition and theoretical basis of the concept

The concept of distributed leadership has been discussed in many scholarly contexts and a variety of attempts to define it have been made in the literature. The concept is not synonymous with shared leadership, nor does it imply that “everyone leads” (Harris, 2008). Fundamental to most conceptualizations of DL is rather the idea that leadership is exercised and shaped through interactions between formal leaders and employees with the sight set towards a common perception of collective influence (see, e.g., Fitzsimons et al. 2011, Gronn 2002, Günzel-Jensen et al. 2018, Spillane 2006). Spillane (2006), for instance, emphasizes that DL is essentially a collective activity. Similarly, Gronn (2002) describes DL as *concertive action* by outlining three patterns of activity that distinguish this type of leadership: (1) spontaneous collaborations where individuals with different skills and competencies jointly contribute with knowledge, (2) the shared role and intuitive understandings that arise when members of an organization “rely on each other and develop close working relationships” (p. 430) and (3) the institutionalization of these practices over time. Central to all these activities is the notion of *conjoint agency*, which refers to interpersonal synergies that can only materialize through mutual cooperation (see also; Currie and Lockett 2011). Clearly, the notion of DL as a fundamentally collective activity challenges traditional views of leadership and implies that formal leaders will provide guidance rather than directives. By extension, this is assumed to enhance influence and promote staff members taking responsibility (Gillberg and Dellve, 2019; Quek et al., 2021). In this study, we draw on the notion of DL as an essentially collective process and use the concept to refer to practices whereby leadership is distributed in everyday work practices. More specifically, we focus on two interrelated aspects of DL: collaborative decision-making and the presence (or lack of) a participatory approach among formal leaders; in other words, practices whereby managers demonstrate trust in employees’ ability to take responsibility, ask for their views and give them opportunities to influence the organization.

### 1.2 Distributed leadership in health and social care settings

While there are many studies on DL, a significant portion of these have been conducted in the educational sector (Bolden, 2011). Due to the fact that the educational sector, unlike the health and social care sectors, chiefly employs one professional group (teachers), more studies on DL in the latter context are needed. This is not least because DL being implemented might entail specific challenges related to the interdisciplinarity of operations (Currie and Lockett, 2011). Additionally, quantitative studies and instruments to assess DL in health- and social care contexts are still scarce and primarily focus on employees’, rather than managers’, experiences (see, e.g., Günzel-Jensen et al. 2018, Jønsson et al. 2016).

Theoretical developments regarding distributed leadership (Gronn, 2002, 2015) suggest that room for assessments and participation in decision-making at operative levels may offer better results in practice. In social and health care contexts, this is believed to be accomplished as more committed and well-informed staff, who are able to take responsibility for the care provided, can better meet the needs of care recipients (Gillberg and Dellve, 2019; Gunnarsdóttir et al., 2018). With regard to institutional and contextual aspects that could potentially facilitate or impede the distribution of leadership, a recent review of collectivistic leadership interventions in health care reveals that aspects such as senior management support, i.e., support from higher management levels in the organization, and time/space to discuss the new approach are crucial for ensuring a successful implementation (De Brún et al., 2019). Other studies underline the importance of communication among team members (Jackson, 2000; Klinga et al., 2016). However, more knowledge concerning the organizational and work team conditions that could potentially impact the inclination of managers to distribute influence and control is still needed (Currie and Lockett, 2011; De Brún et al., 2019). This is not least due to the fact that conditions such as span of control and managerial support are known to play an important role for managers’ work in general (see, eg., Corin and Björk 2016, Wallin et al. 2014). Likewise, factors such as work team engagement, skills development and taking responsibility are well-established predictors of success in organizations’ development work (Andreasson et al., 2016, Gunnarsdóttir et al., 2018).

### 1.3 Aim and hypotheses

**Fig. 1:**
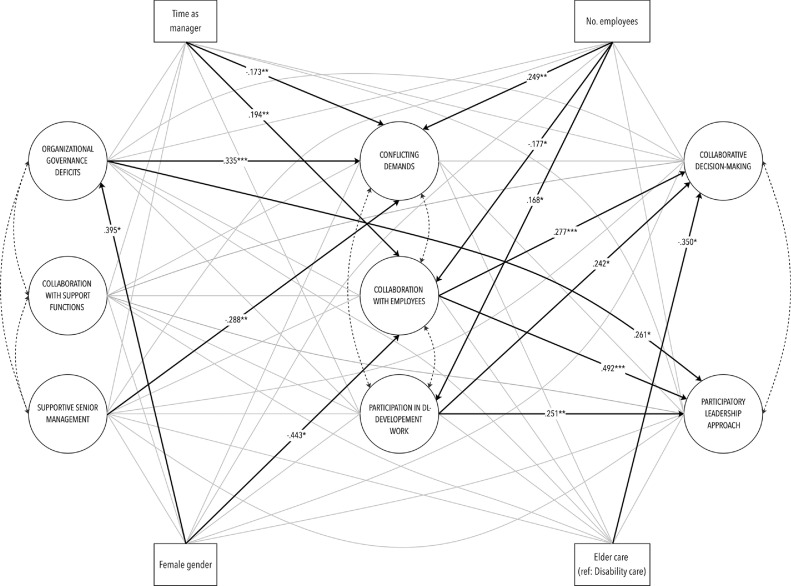
White circles represent latent variables; white rectangles represent observed variables. Bold arrows (incl. estimates) indicate significant associations. Gray lines indicate non-significant associations. Double-headed dotted arrows represent residual covariances (estimates shown in Table 3).

This study aims to identify organizational conditions for distributed leadership in municipal elder- and disability care. More specifically, distributed leadership was hypothesized to be positively associated with first-line managers’ perceptions of a supportive senior management, good collaborations with employees and support functions, as well as active participation in development work aimed at promoting, for instance, trust in the organization (H1). In contrast, distributed leadership was assumed to be negatively associated with organizational governance deficits and conflicting demands in daily work (H2) (Fig. 1).

## 2 Data and methods

### 2.1 Study design

Utilizing survey data from first-line managers working in the elder- and disability care sectors in the municipality of Gothenburg, Sweden, associations between conditions and aspects of distributed leadership were analyzed by means of Structural Equation Modeling (SEM). The study was reported using the STROBE (Strengthening the Reporting of Observational Studies in Epidemiology) guidelines for cross-sectional studies (Von Elm et al., 2007).

### 2.2 Study setting

Gothenburg is the second largest municipality in Sweden and has a highly diverse population, both socio-economically and demographically. The sample contains managers from all district committees. For the last thirty years, Swedish municipalities are responsible for organizing health and social care for the elder and for individuals with disabilities. Legislation and national rights related to social security and employment regulations look the same in all municipalities. Yet, the organization of care services, as well employee working conditions, tend to vary between municipalities. These differences are not only attributable to variation in demographic and financial conditions, but also to more complex processes such as differences in organizational culture.

### 2.3 Sampling, data collection and study sample

Data were collected through an online survey conducted in Gothenburg, Sweden during the winter of 2019–2020 (Oct.–Feb). The target population were all first-line managers in the municipality (*N* = 736, i.e., all entry-level managers (unit managers and group leaders) who worked directly above the non-managerial staff and thus in closest proximity to the delivery of services. Based on these inclusion criteria, a list of potential participants, including their email addresses, was provided by the municipal administration. Thereafter, an invitation including information about the overarching aims of the survey, voluntary participation and anonymity, was sent out. In total, 412 managers responded to the questionnaire (response rate = 56%). Of these, 250 respondents were employed in the elder- and disability care sectors and were thus selected for the present analyses. Accordingly, the managers work in operational areas such as home care services, home health care, nursing homes and personal assistance, activities or home-like living arrangements for individuals with disabilities, including supporting functions within these areas. The vast majority (94.0%) of respondents were unit managers with an average of approximately 30 subordinates, while the rest (6%) were group leaders and had an average of 20 subordinates. Reflecting the fact that women predominate in the elder and disability care sectors, they make up the largest portion of the sample (83.6%). Most managers (95.2%) had undergone post-secondary education and the sample had a mean age of 49 years. The average time spent in a managerial position was a little less than 10 years and just over half of the respondents (59.3%) had ≤ 30 employees (Table 1).

**Table 1:** Demographic characteristics of the study sample (*N* = 250).

	*N* (%)	*M* (SD)
Gender		
Male	41 (16.4)	_
Female	209 (83.6)	_
Age	_	49.3 (9.6)
Managerial responsibility		
Unit manager	235 (94.0)	
Group leader	15 (6.0)	
Post-secondary education		
Yes	220 (95.2)	_
No	11 (4.8)	_
Sector		
Eldercare	143 (57.2)	_
Disability care	107 (42.8)	_
No. subordinates	_	
<30	146 (59.3)	_
>30	100 (40.7)	_
Total number of years working as a manager	_	9.6 (4.7)

### 2.4 Measures

In line with the Structural Equation Modeling (SEM) approach adopted in this study, the two aspects of distributed leadership and their assumed organizational conditions were indicated by latent variables (also commonly referred to as latent factors). These variables were estimated by means of Confirmatory Factor Analysis (CFA) and comprise the covariance between a set of observed variables (or manifest indicators) (Brown, 2015). An overview of all focal study variables and their indicators can be found in Table 3.

#### 2.4.1 Distributed leadership

The first aspect of distributed leadership, *collaborative decision-making* (Cronbach's *α* = 0.71), includes two items obtained from the Managerial Ethical Profile (MEP) (Casali, 2011). This instrument entails questions concerning operative decision-making, such as “How important is *X* when you make decisions to live up to the requirements resulting from your position in the organization.” The two items used pertain to the involvement of employees and other concerned parties. Responses were given on a scale ranging from 1 “not at all important” to 5 “extremely important.” The second aspect, *participatory leadership approach* (Cronbach's *α =* 0.70), includes three items obtained from the Gothenburg Manager Stress Inventory (GMSI) (Eklöf et al., 2010). The questions in this instrument concerning leadership are phrased so that respondents are asked to assess aspects of their own leadership, e.g., the extent to which they exhibit trust in employees’ ability to take responsibility, ask for their views and offer them opportunities to influence the organization. Responses were given on scales ranging from 1 “not at all true” to 5 “completely true”.

#### 2.4.2 Organizational conditions

The study includes measures of six organizational conditions. The first five also comprise sets of items retrieved from GMSI (Eklöf et al. 2010, see above). First, *supportive senior management* (Cronbach's *α =* 0.91) consists of five items. It aims to capture the level of support received by managers from senior management levels as well as the extent to which senior managers take responsibility for ensuring a sustainable work situation. Second, *organizational governance deficits* (Cronbach's *α =* 0.79) contains four items and is used to indicate the perceived prevalence of unclear procedures as well as a lack of clarity concerning the division of responsibilities in the organization. Third, *collaboration with employees* (Cronbach's *α =* .88) comprises five items. It aims to reflect managers’ perceptions of mutual trust and responsibility between them and their employees, as well as the extent to which they are able to engage in favorable collaborations with knowledgeable employees. Fourth, *conflicting demands* (Cronbach's *α =* .88), comprising five items, is intended to capture the prevalence of conflicting demands/tasks in managers’ daily work. Finally, *collaboration with support functions* (Cronbach's *α =* 0.88) includes three items and aims to reflect managers’ perceptions of having trusting collaborations with organizational support functions such as administration or human resources. The sixth latent variable, *active participation in DL development work*, contains three items developed for the purposes of the survey. Managers were asked to assess the extent to which they, during the past year, had driven or actively participated in development work aimed at, for instance, increasing employee participation in operative decision-making or promoting trust in the organization. Responses were given on scales ranging from 1 “no, not at all” to 5 “yes, to a very high degree”.

#### 2.4.3 Covariates

To control for potentially confounding effects, a number of demographic variables were included in the structural model. *Gender* (Female = 1, Male = 2) was indicated by a dichotomous variable, while *total number of years working as a manager* and *number of employees* were both measured on continuous scales (Table 1).

### 2.5 Data analysis

The study applied SEM with robust full information maximum likelihood (FIML) estimation (Rhemtulla et al., 2012). Following standard CFA/SEM procedures, the analysis was carried out in two steps. First, CFA was employed to estimate two measurement models comprising the latent variables, one including the six organizational condition variables and the other including the two DL variables. This was done in order to confirm that the manifest variables were linked to the latent factors, as hypothesized. Second, a full structural model, including both latent variables and covariates, was estimated to test the main hypotheses. As the directions of the relationships between, for instance, supportive management and organizational governance deficiencies, or collaboration with employees and conflicting demands, are theoretically difficult to assess, no regression paths were specified between these. However, since these phenomena are likely to be related, mutually reinforce each other and share common causes, their residuals were allowed to covary (see Fig. 1, residual covariances indicated by double-headed dotted arrows). In both stages of the analysis procedure, we relied on the following fit indices and cutoff criteria to evaluate model fit: a Root Mean Square Error of Approximation (RMSEA) of close to 0.06 or below, a Tucker–Lewis Index (TLI) of close to 0.95 or above and a Standardized Root Mean Square Residual (SRMR) of less than 0.08 (Hu and Bentler, 1999). Model χ2 is also reported; however, since it is known to be sensitive to sample size (Browne and Cudeck, 1992; Byrne, 2013), significant values were not considered to be a major concern. Data management, data cleaning and descriptive statistics were carried out in STATA version 16.0, and the CFA/SEM analyses were conducted in MPlus version 8.1. In the descriptive analyses we report percentages or averages with standard deviations in brackets. As to the CFA/SEM results, standardized point estimates are reported alongside 95% confidence intervals in brackets.

### 2.6 Ethical approval

The present research project was approved by the Central Ethical Review Board (Dnr: 2019–02,934).

## 3 Results

### 3.1 Descriptive analyses

On scales ranging from 0 to 10, the managers’ average ratings of their own DL practices were high (7.2 [1.7] and 7.2 [2.0], respectively). In contrast, their engagement in DL development work was relatively low (4.6 [2.7]). As to the other organizational conditions, also assessed on scales ranging from 0 to 10, they rated their collaboration with both support functions (6.5 [2.2]) and employees (7.0 [2.1]) higher than the support they themselves received from senior management levels (5.1 [2.6]). Furthermore, although the managers reported relatively low levels of organizational governance deficits (4.4 [2.0]), the results indicate that they are often faced with conflicting demands (6.7 [2.1]). Finally, all organizational conditions except governance deficits were significantly correlated with either one or both aspects of DL in the bivariate analyses (Table 2).

**Table 2:** Descriptive statistics, reliability and pairwise correlations for the focal measures.

	M (SD)	Cronbach's α	(1)	(2)	(3)	(4)	(5)	(6)	(7)	(8)
(1) Collaborative decision-making^a^	7.2 (1.7)	0.71	1.00							
(2) Participatory leadership approach^a^	7.2 (2.0)	0.70	**0.38**	1.00						
(3) Active participation in DL development work^a^	4.6 (2.7)	0.82	**0.28**	**0.24**	1.00					
(4) Organizational governance deficits^a^	4.4 (2.0)	0.79	−0.08	−0.07	**−0.15**	1.00				
(5) Conflicting demands^a^	6.7 (2.1)	0.85	**−0.13**	−**0.20**	**−0.25**	**0.51**	1.00			
(6) Supportive senior management^a^	5.1 (2.6)	0.91	**0.15**	**0.14**	**0.25**	**−0.46**	**−0.43**	1.00		
(7) Collaboration with support functions^a^	6.5 (2.2)	0.68	0.08	**0.13**	**0.16**	**−0.35**	**−0.25**	**0.38**	1.00	
(8) Collaboration with employees^a^	7.0 (2.1)	0.88	**0.26**	**0.43**	**0.18**	**−0.26**	**−0.40**	**0.20**	**0.15**	1.00

Note:.

a = Index, range: 0–10. Pairwise Spearman's rank correlation coefficients. Significant (*p*<0.05) estimates in bold print.

### 3.2 Measurement models

First, a measurement model including all six latent condition variables was estimated by means of CFA. Factor loadings were generally high (the vast majority > 0.6) and ranged from 0.527 to 0.909. As the modification indices (MIs) and the corresponding estimates of expected parameter change (EPCs) suggested that two residuals should be allowed to covary (SM1 with SM2 and CS1 with CS2), the default constraints on these parameters were relaxed. After these adjustments, the following fit indices were obtained and the model fit was considered satisfactory: χ^2^ (df) = 441.547 (258), RMSEA [90% CI] = 0.053 [0.045–0.062], TLI = 0.927, SRMR = 0.056 (Table 3). Second, a measurement model including the two latent DL variables was estimated. Again, factor loadings were high (all ≥ 0.6) and ranged from 0.600 to 0.919. As indicated by the following fit indices, the model fit was considered satisfactory and no further adjustments were needed: χ^2^ (df) = 1.529 (4), RMSEA [90% CI] = 0.000 [0.000–0.058], TLI = 1.028, SRMR = 0.012 (Table 3).

**Table 3:** Measurement models.

	ORGANIZATIONAL PRECONDITIONS^a^^,^^b^			DISTRIBUTED LEADERSHIP^b^		
	Coef^c^	95% CI	*P*	Coef^c^	95% CI	*p*
SUPPORTIVE SENIOR MANAGEMENT (SM)						
Senior management shows a genuine interest in what I do and the problems I have as a manager/leader (SM1)	0.721	0.654–0.787	^⁎⁎⁎^	–	–	–
I have good support from my superiors when it comes to personnel and work environment issues (SM2)	0.759	0.699–0.820	^⁎⁎⁎^	–	–	–
Senior management is just as concerned to ensure good working conditions as to achieve other organizational goals (SM3)	0.871	0.826–0.917	^⁎⁎⁎^	–	–	–
I trust that the senior management, if necessary, will help me get a work situation that I can handle (SM4)	0.909	0.878–0.940	^⁎⁎⁎^	–	–	–
Senior management levels in the organization take responsibility for clearly showing what level of quality is possible with the resources that my unit receives (SM5)	0.751	0.682–0.821	^⁎⁎⁎^	–	–	–
ORGANIZATIONAL GOVERNANCE DEFICITS (GD)						
Decisions made higher up in the organization are very difficult or impossible to implement at your unit (GD1)	0.754	0.681–0.827	^⁎⁎⁎^	–	–	–
You find it difficult to overview the decision-making processes in the organization (GD2)	0.729	0.649–0.810	^⁎⁎⁎^	–	–	–
You find it difficult to get a clear picture of what you as a manager is responsible for (GD3)	0.735	0.656–0.815	^⁎⁎⁎^	–	–	–
You have to adapt to strict demands for uniformity (GD4)	0.585	0.476–0.694	^⁎⁎⁎^	–	–	–
COLLABORATION WITH EMPLOYEES (CE)						
I feel that my employees want to take responsibility in their work (CE1)	0.758	0.692–0.824	^⁎⁎⁎^	–	–	–
I feel that employees have valuable knowledge that makes my work easier (CE2)	0.853	0.808–0.898	^⁎⁎⁎^	–	–	–
My employees are confident in their professional roles (CE3)	0.799	0.744–0.854	^⁎⁎⁎^	–	–	–
I feel that I can trust that employees follow my instructions (CE4)	0.888	0.842–0.934	^⁎⁎⁎^	–	–	–
Processing work-related problems and questions together with employees works well (CE5)	0.733	0.654–0.812	^⁎⁎⁎^			
CONFLICTING DEMANDS (CD)						
You cannot devote enough time to organizational development (CD1)	0.753	0.676–0.830	^⁎⁎⁎^	–	–	–
You have to spend too much time on administrative work (CD2)	0.731	0.651–0.812	^⁎⁎⁎^	–	–	–
You have difficulties finding the time to meet your employees to discuss issues that arise in the daily work (CD3)	0.741	0.671–0.810	^⁎⁎⁎^	–	–	–
There are situations when you feel that you have to do several different things at the same time (CD4)	0.603	0.509–0.697	^⁎⁎⁎^	–	–	–
Tensions arise between administrative work, organizational development and contact with employees (CD5)	0.831	0.777–0.885	^⁎⁎⁎^	–	–	–
COLLABORATION WITH SUPPORT FUNCTIONS (CS)						
I have a trusting collaboration with the human resources department (CS1)	0.527	0.370–0.684	^⁎⁎⁎^	–	–	–
I have a trusting collaboration with administrative support functions (CS2)	0.675	0.551–0.799	^⁎⁎⁎^	–	–	–
I have a trusting collaboration with resource persons (organizational developers, lean coaches, improvement leaders or the like) in organizational development work (CS3)	0.723	0.592–0.855	^⁎⁎⁎^	–	–	–
ACTIVE PARTICIPATION IN DL DEVELOPMENT WORK (DW)						
… to organize for increased trust between unit managers and employees (DW1)	0.860	0.779–0.941	^⁎⁎⁎^	–	–	–
… to organize for increased trust between support functions and unit managers (DW2)	0.722	0.628–0.817	^⁎⁎⁎^	–	–	–
… to increase employee participation in operative decision-making (DW3)	0.719	0.621–0.817	^⁎⁎⁎^	–	–	–
COLLABORATIVE DECISION-MAKING (CDM)						
Importance assigned to: Involving employees in the decision-making (CDM1)	–	–	–	0.919	0.724–1.114	***
Importance assigned to: Giving all parties an opportunity to have a say in the decision-making process (CDM2)	–	–	–	0.600	0.433–0.767	***
PARTICIPATORY LEADERSHIP APPROACH (PA)						
I ask employees for their views and ideas (PA1)	–	–	–	0.662	0.556–0.768	***
I give employees the opportunity to influence the formation and future of the organization (PA2)	–	–	–	0.667	0.560–0.773	***
I show employees that I trust their ability to take responsibility (PA3)	–	–	–	0.662	0.545–0.779	***
*N*	250			245		
χ^2^ (df)	441.547 (258)			1.529 (4)		
TLI	0.927			1.028		
RMSEA [90% CI]	0.053 [0.045–0.062]			0.000 [0.000–0.058		
SRMR	0.056			0.012		

(a) The model also includes the following, non-default, residual covariances (estimates excluded for clarity reasons but are available upon request): SM1 ⇔ SM2 & CS2 ⇔ CS4.

(b) For 25 of the total 30 variables, the rate of missing was below 3%. The highest rate of missing observed was 12.8% (DW2).

(c) Standardized coefficients, *p < 0.05. **p < 0.01. ***p < 0.001.

### 3.3 Structural model

The full structural model concurrently tested our two hypotheses and demonstrated a good fit to the data: χ^2^ (df) = 732.209 (463), RMSEA [90% CI] = 0.049 [0.042–0.056], TLI = 0.903, SRMR = 0.053. In accordance with H1, the model posited that both aspects of distributed leadership (i.e., collaborative decision-making and a participatory leadership approach) would be positively associated with a supportive senior management, collaboration with employees and support functions, as well as active participation in DL development work, also when controlling for confounders (gender, sector, number of employees and total number of years working as a manager) (Fig. 1). This hypothesis was only partially confirmed by the data. In line with H1, both aspects of distributed leadership were shown to be significantly associated with collaboration with employees (CDM = 0.277 [0.122–0.432]; PA = 0.492 [0.346–0.637]) and active participation in DL development work (CDM = 0.242 [0.039–0.446]; PA = 0.251 [0.103–0.398]). Contrary to our expectations, no significant associations between distributed leadership and a supportive senior management or collaboration with support functions were observed. Furthermore, neither collaboration with employees nor participation in DL development work (i.e., the conditions shown to be associated with distributed leadership) was significantly related to any of the other latent condition variables (organizational governance deficits, collaboration with support functions or a supportive senior management). Although no regression path was drawn between conflicting demands and collaboration with employees (see above), it should be noted that their residual covariance was significant (−0.249 [−0.389 to −0.109]). Thus, even though the direction of the relationship between these latent variables was not specified, the significant covariance of their residuals suggests that these phenomena are related and that they share something that could not be captured by the included predictors. With respect to H2, we hypothesized that both aspects of distributed leadership (i.e., collaborative decision-making and a participatory leadership approach) would be negatively associated with organizational governance deficits and conflicting demands, also when controlling for confounders. Partially in accordance with H2, organizational governance deficits were shown to be significantly, albeit positively, associated with a participatory leadership approach (0.261 [0.032–0.491]) but not with collaborative decision-making. Furthermore, even though the experience of conflicting demands was significantly predicted by both organizational governance deficits and a lack of a supportive senior management, it was not significantly related to distributed leadership.

As to the included confounders, managers in eldercare rated their practice of collaborative decision-making significantly lower compared to their counterparts in disability care (−0.350 [−0.644 to −0.56]), while none of the other confounding variables were significantly associated with distributed leadership. However, both collaboration with employees (−0.177 [−0.315 to −0.039]) and participation in DL development work (0.168 [0.012–0.323] were significantly associated with the number of employees, albeit in opposite directions. In addition, female managers rated their collaborations with subordinates lower compared to male managers (−0.443 [−0.788 to −0.099]). The results further suggest that having more positive perceptions of one's collaboration with employees is positively related to the number of years working in a managerial position (0.194 [0.073–0.314]).

## 4 Discussion

While several studies have previously identified potential positive outcomes of DL (Boak et al., 2015; De Brún et al., 2019; Greenfield et al., 2009; McKee et al., 2013; Quek et al., 2021), the present study contributes to this field by identifying important organizational and work team conditions for the distribution of leadership among first-line managers in the elder- and disability care sectors. Overall, the results suggest that collaboration with responsible and trained staff, as well as active participation in development work aimed at promoting organizational trust and employee participation, were the most important conditions for managers to distribute their leadership. In turn, both of these conditions were related to the number of subordinates in the sense that the more employees, the more active engagement in DL development work but the fewer well-functioning collaborations between managers and subordinates. Furthermore, better collaborations with subordinates were reported among male managers as well as among managers with longer managerial experience. Somewhat unexpectedly, the results also suggest that managers’ perceptions of organizational governance deficits are related to a more participatory leadership approach.

### 4.1 Collaboration and responsibility

The results highlight that good and trusting collaborations with responsible and competent staff are key for managers’ ability and willingness to distribute leadership. Considering that, at its core, DL is a collective activity enabled through interaction and mutual cooperation (see, e.g., Fitzsimons et al. 2011, Gronn 2002, Günzel-Jensen et al. 2018, Spillane 2006), these results are not surprising. In fact, as underlined by Gronn (2002), the concertive action characterizing DL is based on the presence of spontaneous and synergetic collaborations in which all parties jointly contribute, exchange knowledge and, over time, develop trusting working relationships (see also Currie and Lockett 2011). In this study, collaboration with employees has primarily been conceptualized as a *precondition* for DL. In practice, however, it is reasonable to assume that well-functioning collaborations and distributed leadership practices mutually influence and reinforce each other. In line with this, previous studies have shown that communication among team members (Jackson, 2000; Klinga et al., 2016) and a team environment characterized by, for instance, social support and a shared purpose (Carson et al., 2007) are crucial for ensuring success when implementing more collectivistic leadership approaches (see also, De Brún et al. 2019). At the same time, factors such as enhanced communication, trust and support can be considered important *outcomes*, or byproducts, of such interventions (Boak et al. 2015, see also De Brún et al. 2019). Hence, while positive collaborations with knowledgeable and responsible employees are likely to facilitate the materalization of DL, the relationship between these workplace dynamics is most likely characterized by reciprocal and positive exchanges.

### 4.2 Organizational deficits and conflicting demands

A recent review of collectivistic leadership interventions in health care suggests that senior management support is crucial for ensuring success in the implementation process (De Brún et al., 2019). However, in our controlled analysis, neither support from senior management levels nor constructive collaborations with organizational support functions was significantly associated with DL. However, our findings suggest that organizational governance deficits (i.e., managers’ perceptions of unclear procedures and a lack of clarity concerning responsibilities in the organization) are in fact related to *more* participatory leadership approaches. While this might appear somewhat contradictory, a possible interpretation is that perceived deficits at higher levels in the organization increase managers’ propensity to delegate or, in the worst case, ‘dump’ responsibilities on their employees. However, considering the manifest items that are included in our measure of participatory leadership (Table 3), it appears more reasonable to assume that perceived deficits encourage managers to turn to their employees for guidance and advice. In addition, another contributing explanation to these results could be that organizational governance deficits create increased room for formal leaders to form their own leadership practices (e.g., by means of distributing control) and thereby develop closer working relations and collaborations with their team (see, e.g., Andreasson et al. 2016). Naturally, this does not suggest that governance deficits are desirable, but it might imply that implementing distributed leadership necessitates a certain “room for maneuver” among first-line managers (Gillberg and Dellve, 2019). Finally, while no direct relationship between conflicting demands and DL could be confirmed in the controlled model, it should be emphasized that such demands appear to be negatively related to collaboration with staff and were predicted by both organizational governance deficits and poor support from senior management levels.

#### 4.2.1 Strengths and limitations

The principal strengths of the study include the use of validated survey questions and the structural equation modeling approach. Owing to the latter, we were able to concurrently test the relationships between a wide range of latent outcomes and predictors. Thereby, the errors in measurement normally associated with using additive indices were reduced and the complexity and multidimensional nature of intra-organizational relations could be addressed. However, our sample is limited to the municipality of Gothenburg. Although Gothenburg is demographically diverse and the sample contains managers from all district committees, our results are not necessarily generalizable to other Swedish municipalities. This is because the organization of care services as well employee working conditions tend to vary between these. Additionally, because of the moderate response rate and limited sample, our results must be interpreted with caution. First, it cannot be ruled out that non-significant associations are attributable to a lack of power. Second, we did not examine specific subgroups (e.g., managers in different areas of operation), nor potential interaction effects as it would significantly increase model complexity. Finally, the cross-sectional design implies that no conclusions concerning causality or its potential direction can be drawn from the present study.

#### 4.2.2 Conclusion and directions for future studies

In line with the notion of DL as a “collective activity”, realized in the interaction between managers and their subordinates, this study underlines that trusting collaborations with responsible and knowledgeable employees are key for its practical implementation. Nevertheless, the relationship between DL, and the work team relations through which it is enabled, is unlikely to be unidirectional. It is instead reasonable to assume that these workplace dynamics mutually reinforce each other, which is why future studies should seek to provide further insights as to whether and how these and additional organizational conditions relate to other aspects of DL in health and social care settings. Likewise, since some studies indicate that employees at the operational level may in fact be unwilling to shoulder greater responsibilities and consider wage levels and decent working hours more important than influence (Gillberg and Dellve, 2019), future studies should attempt to further explore the impact of DL on work team dynamics, including potentially negative experiences among employees. In turn, such studies depend on the development of validated instruments that conceptually and empirically distinguish different DL practices and thereby enable explorations of their potential consequences for managers and employees (Jønsson et al., 2016). Finally, the results of the present study suggests that managers’ experiences of poor organizational governance could actually promote certain DL practices. This could potentially be because such deficits encourage leaders to seek support and guidance from their employees and/or implies an increased “room for maneuver” among formal leaders. However, more research further exploring these potential mechanisms is needed.

#### 4.2.3 Practical implications

The findings of this study underline that organizational efforts to promote collaboration and trust between first-line managers and their employees are key for ensuring a successful implementation of distributed leadership practices in elder- and disability care settings. Furthermore, since managers’ inclination to delegate and distribute control appears to be related to their perceptions of employees’ willingness to shoulder responsibility, as well as to their “professional confidence”, continuous skills development for those working the closest to care recipients is also of utmost concern. Against the backdrop of challenges such as difficulties to recruit trained staff and high turnover in welfare organizations, it is, however, also crucial to ensure that employees who choose to shoulder increased responsibilities and/or undergo further training, are rightfully compensated for their efforts.

## “What is already known”

Distributed leadership challenges traditional views on leadership since it is essentially a “collective activity” realized in the interaction between managers and their employees.

While there are many studies on distributed leadership, a significant portion of these have been conducted in the educational sector.

Although distributed leadership is likely to, for instance, have a positive impact on the quality of care and improve job satisfaction, little is known regarding its organizational precursors in health and social care settings.

## “What this paper adds”

Owing to its methodological approach, this study makes a contribution by offering an empirical conceptualization of distributed leadership and by concurrently testing its relationship with a wide range of organizational conditions in elder- and disability care contexts.

In line with the notion of distributed leadership as a “collective activity”, the findings underline that trusting collaborations with responsible and knowledgeable employees play a key role in its practical implementation.

The findings also suggest that managers’ experiences of poor organizational governance may in fact promote certain distributed leadership practices, since these potentially encourage leaders to seek support and guidance from their employees and imply an increased “room for maneuver”.

## Uncited links

Table 4

**Table 4:** Structural model.

	Structural model^a^^,^^b^		
	Coef.^c^	95% CI	*p*^c^
SUPPORTIVE SENIOR MANAGEMENT (SM) ⇒			
Collaboration w. employees	0.105	−0.073–0.284	ns
Conflicting demands	−0.288	−0.458- −0–118	**
Active participation in DL development work	0.190		ns
Collaborative decision-making	0.082	−0.148–0.313	ns
Participatory approach	0.091	−0.093–0.274	ns
ORGANIZATIONAL GOVERNANCE DEFICITS (GD) ⇒			
Collaboration w. employees	−0.137	−0.306–0.032	ns
Conflicting demands	0.335	0.173–0.496	***
Active participation in DL development work	−0.126		ns
Collaborative decision-making	0.105	−0.166–0.376	ns
Participatory approach	0.261	0.032–0.491	*
COLLABORATION WITH SUPPORT FUNCTIONS (CS) ⇒			
Collaboration w. employees	0.112	−0.083–0.307	ns
Conflicting demands	−0.082	−0.270–0.106	ns
Active participation in DL development work	0.001		ns
Collaborative decision-making	0.037	−0.194–0.267	ns
Participatory approach	0.173	−0.043–0.390	ns
CONFLICTING DEMANDS (CD) ⇒			
Collaborative decision-making	−0.034	−0.287–0.219	ns
Participatory approach	−0.018	−0.234–0.199	ns
COLLABORATION W. EMPLOYEES (CE) ⇒			ns
Collaborative decision-making	0.277	0.122–0.432	***
Participatory approach	0.492	0.346–0.637	***
ACTIVE PARTICIPATION IN DL DEVELOPMENT WORK (DW)			
Collaborative decision-making	0.242	0.039–0.446	*
Participatory approach	0.251	0.103–0.398	**
SM_res_ ⇔ GD_res_	−0.543	−0.679- −0.408	***
SM_res_ ⇔ CS_res_	0.505	0.348–0.663	***
GD_res_ ⇔ CS_res_	−0.478	−0.651- −0.305	***
CD_res_ ⇔ CE_res_	−0.249	−0.389- −0.109	***
CD_res_ ⇔ DW_res_	−0.136	−0.318–0.045	ns
CE_res_ ⇔ DW_res_	0.184	0.032–0.336	*
CDM_res_ ⇔ PA_res_	0.346	0.118–0.574	**
N	243		
χ^2^ (df)	732.209 (463)		
TLI	0.906		
RMSEA [90 CI]	0.049 |0.042–0.056]		
SRMR	0.053		

(a) Adjusted for: Gender, total number of years working as a manager and number of employees (estimates shown in Fig. 1).

(b) The model also includes the following, non-default, residual covariances (estimates excluded for clarity reasons but are available upon request): SM1 ⇔SM2 & CS2 ⇔ CS4.

(c) Fully standardized coefficients, *p < 0.05, **p < 0.01, *** *p* < 0.001.

## Declaration of Competing Interest

The authors declare that they have no known competing financial interests or personal relationships that could have appeared to influence the work reported in this paper.
